# Development and evaluation of a personalised sleep care plan on child and adolescent in-patient mental health wards

**DOI:** 10.1192/bjb.2024.41

**Published:** 2025-08

**Authors:** Kirstie N. Anderson, Rod Bowles, Christine Fyfe, Ron Weddle, Patrick Keown

**Affiliations:** 1 Newcastle upon Tyne Hospitals NHS Foundation Trust, Newcastle upon Tyne, UK; 2 Newcastle University, Newcastle upon Tyne, UK; 3 Cumbria, Northumberland, Tyne and Wear NHS Foundation Trust, Newcastle upon Tyne, UK

**Keywords:** Education and training, sleep–wake disorders, risk assessment, psychiatric nursing, mental health services

## Abstract

**Aims and method:**

The study evaluated a package of measures to improve sleep on psychiatric wards admitting patients from children and young people's services (CYPS). Sleep disturbance has significant impact on adolescent mental health, and in-patient wards can directly cause sleep disturbance, independent of the problem that led to admission. We developed a CYPS-specific package (TeenSleepWell) that promoted a better sleep environment, enhanced staff education about sleep, screened for sleep disorders, and raised awareness of benefits and side-effects of hypnotics. This included personalised sleep care plans that allowed a protected 8 h sleep period when safe.

**Results:**

Evaluation over 2 years showed enhanced in-patient care: 57% of patients were able to have a protected sleep period. There was no increase in adverse events and there was a decrease in hypnotics issued.

**Clinical implications:**

Improving sleep during in-patient CYPS admissions is possible and personalised sleep care plan should be a care standard.

Sleep disruption affects physical and mental health and is an independent risk factor for suicide.^1^ In addition, sleep disorders such as insomnia, delayed sleep phase syndrome and restless legs syndrome are common among adolescents but remain underdiagnosed despite effective therapies.^2,3^ Children and adolescents in hospitals have sleep disturbance due to high noise levels, light at night and overnight nursing observations.^4^ It is therefore important to optimise sleep in mental health units admitting patients from children and young people's services (CYPS), to help recovery. Wards should be conducive to restful sleep and an in-patient stay should be an opportunity to detect and manage sleep disorders.

In hospital, there is a need to balance safe levels of observation overnight to reduce the risk of harm against the disruption of sleep caused by nursing observations. During an admission, risk will not remain static. However, many CYPS mental health wards have hourly observations day and night throughout an admission. This practice has been challenged, given that sleep disturbance caused by physical checks increases agitation.^5^ Use of hypnotics on adolescent wards occurs despite the side-effects of medications.^6^

Previous work on our mental health trust's adult wards demonstrated high noise levels at night, sleep disturbance due to nursing observations and many with sleep disorders, including obstructive sleep apnoea.^7^ The SleepWell programme increased education about sleep and sleep disorders, decreased overnight noise and allowed protected overnight sleep when safe. This reduced the use of hypnotics and enhanced care without increasing adverse events.^8^ Hospital engagement and observation policy was changed to allow trust-wide implementation. CYPS units were excluded from the initial pilot. We have therefore evaluated a package of measures specific to sleep on CYPS wards.

## Method

**Fig. 1:**
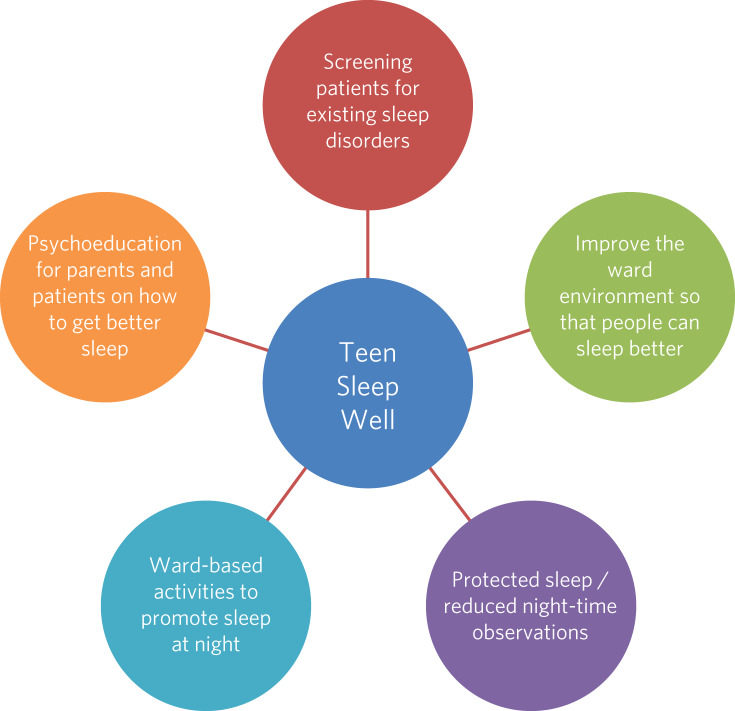
The TeenSleepWell measures to improve sleep on children and young people's mental health wards.

A pilot study was carried out on four CYPS wards within a large mental health trust to evaluate a package of measures to improve sleep (Fig. 1). The units were: intellectual disability (also known as learning disability in UK health services); secure; and general adolescent in-patient (with an intensive care unit); in total there were 40 beds for patients aged 12 to 18 years. Outcomes were evaluated over 2 years. We are unaware of any similar sleep improvement programmes in the UK.

This was a registered quality improvement project that did not require ethical approval but did have prior approval from the trust management board. Staff and patients gave informed consent where statements concerning feedback on the programme occur in the text.

There were nominated sleep leads across the four units and age-specific educational resources about sleep were developed within a trust sharepoint. All staff working on the units underwent training in normal sleep using a standardised slide set developed by K.N.A. which explained the role of sleep disturbance in mental health, adolescent sleep disorders and the benefits and side-effects of hypnotics. Understanding that the adolescent circadian rhythm predisposes to later sleep onset was key information given to staff, carers and patients. Staff on wards supported patients to access outdoor light for at least 1 h per day to improve daytime alertness and sleep whenever possible. The importance of darkness at night was explained, with wind-down and decrease of screen light from TVs or computers or devices in the hour before bedtime.

To detect specific sleep disorders, wards used a validated Restless Legs Syndrome screening question.^9^ Circadian rhythm disorders such as delayed sleep phase syndrome were differentiated from insomnia by using simple sleep logs and nursing observations of bed and rise time. Recommended noise levels on hospital wards at night is below 35 dB.^10^ Opportunities to reduce noise used dedicated support from the trust's estates department. Each sleep lead identified noise points on their ward at night during initial meetings in 2020. Solutions included vibrating ward phones, soft-closing bins and television turned down or off after 22.00 h. The role of melatonin in helping sleep onset but being suppressed by light at night was explained, alongside the need for consistent timing of prescribed melatonin. Knowledge of the patient's habitual bedtime and also reviewing ongoing need and benefit of hypnotic medication were documented in a sleep care plan.

There was patient and carer input throughout. Key stakeholders included pharmacy, estates, the paediatric sleep service and ward managers, who all had an input. Meetings were held every 3 months for development of the package of measures, and implementation and progress reports across all units continued for 2 years. Total issues of the hypnotic medications melatonin, promethazine and zopiclone were measured prior to starting and then measured over the subsequent 2 years (no other hypnotics were issued). After a minimum period of 72 h following admission, and after risk assessment, a multi-disciplinary team could agree to a protected 8 h sleep period without mandatory hourly checks between 23.00 h and 07.00 h. This was longer than on adult wards because the average sleep need between 12 and 18 years of age is 8–10 h.^11^ Night shift staff discussed who was on the protected sleep period at daily handover but could override decisions at any time if they had concerns about a patient's physical or mental health. Every 3 months, data were reported recording the number of patients considered safe for a protected sleep period, alongside a review of adverse events (day or night) measured using standard trust reporting systems. The number of patients appropriate for protected sleep was reported by each sleep lead. Feedback from staff and patients was collected during semi-structured interviews in December 2022.

## Results

All wards successfully implemented the TeenSleepWell package between December 2020 and November 2022. It was then maintained as a service improvement across all pilot wards (40 beds in total). This was a popular intervention, maintained despite staffing difficulties throughout the COVID-19 pandemic. The educational resources and training delivered to all ward staff throughout the implementation were considered key to allaying initial concerns about adjusting night observations and how to identify and manage risk. Review of the monthly incident-reporting dashboards for the year before the pilot (January to the start of December 2020) and then for the 2 years of the pilot confirmed that this was a safe intervention. Specifically, there were no reported adverse events or episodes of self-harm during a period of protected sleep during the 2 years of the pilot. A review of ward sleep care plans showed that 57% were appropriate for protected sleep in December 2022. Many with ongoing physical and mental health needs still required overnight observations but personalised sleep plans were welcomed by all, as the overnight observations were recognised to be a source of tension at night.

The total amount of hypnotic medication issued to each ward was measured over the year prior to starting TeenSleepWell and then for the 2 years of the study (Table 1). Ward reconfiguration led to a decrease in total beds during the 2-year evaluation so the total issues per bed were calculated. During the 2 years of TeenSleepWell, there was no change in the amount of melatonin issued per bed and this remained by far the most frequently used hypnotic. The amount of zopiclone issued per bed decreased. Promethazine use was unchanged (this excluded promethazine used within acute sedation protocols). The decreased use of zopiclone was maintained after the formal evaluation of the pilot was completed in December 2022.

**Table 1:** Total hypnotic medication issued on four children and young people's mental health wards^a^

Year	Promethazine	Zopiclone	Melatonin
2020	66 issues Total cost £156.70 2.2 issues per bed	67 issues Total cost £23.55 2.2 issues per bed	197 issues Total cost £5949 6.7 issues per bed
2021	143 issues Total cost £174.99 4.77 issues per bed	73 issues Total cost £17.07 2.4 issues per bed	261 issues Total cost £4829 8.7 issues per bed
2022	71 issues Total cost £689 2.36 issues per bed	12 issues Total cost £4.72 0.4 issues per bed	161 issues Total cost £1819 6.7 issues per bed
2023 (6 months)	35 issues Total cost £217.73 1.46 issues per bed	2 issues Total cost £0.92 0.08 issues per bed	37 issues Total cost £701 1.54 issues per bed

a. There was ward reconfiguration and a decrease in total bed numbers for a period during the 2-year evaluation, so the total issues per bed were calculated, alongside the cost of the medication.

Anonymised staff and patient feedback was collected across all wards in December 2022 by a patient and carer lead in semi-structured interviews in which participants were asked five questions about ward sleep: their opinion on the benefit of TeenSleepWell; whether any patient preferred hourly checks; whether the ward valued sleep; whether the ward was peaceful in the evening; and whether anything further could be done to improve overnight sleep. At least four staff members across day and night staff on each unit contributed feedback. We show here examples of responses when participants were asked whether the programme was liked and whether there were concerns about changing observations:

‘I agree with TeenSleepWell. I don't like disturbing people's sleep’ (staff member).

‘Absolutely brilliant idea’ (staff member).

‘It helps a lot especially with young people with heightened senses, as even the key turning the viewing window is enough to disturb some people's sleep’ (staff member).

‘Yes it's good to have good sleep’ (patient).

‘Great idea’ (patient).

‘I would say the ward is usually peaceful at night’ (patient).

None of the ward leads or patients raised concerns. The programme was seen as beneficial to patients and received subsequent approval for implementation as a trust-wide service improvement. A common theme in staff feedback was that a protected sleep period was not appropriate for all patients and always required a personalised risk assessment, given that some had physical or mental health needs requiring regular overnight nursing input. Another common theme was that staff new to the pilot wards needed support and training to be reassured that the change to the overnight nursing policy was safe. Regular meetings were felt to be key to supporting staff, alongside support from senior nursing leads. However, for many patients and carers TeenSleepWell was seen as supportive of discharge planning, when overnight observation would not be carried out, and supportive of planned weekend leave.

## Discussion

Despite a clear understanding of the impact of sleep disturbance on adolescent mental health,^1^ there has been very little work that has directly studied the barriers and facilitators of sleep and circadian rhythm on CYP mental health units. We describe the safe implementation of a package of measures to improve sleep and circadian rhythm on CYP wards and are not aware of any other previous similar service improvement in this area.

Previous work studying children and adolescents in hospitals has shown significant sleep and circadian rhythm disturbance and reduction in total sleep time, highlighting frequent overnight nursing observations and noise on the wards as modifiable risk factors, alongside low light levels in the day and excess light at night.^4^ The same group showed that a successful multimodal approach to improving child and adolescent sleep was possible in physical health wards – the Sleep for Health in Hospital programme (https://www.piernetwork.org/shh.html). However, there has not been a similar programme carried out in CYPS mental health units. Within UK CYPS units, it remains standard practice to have fixed and frequent (typically not less than hourly) physical checks throughout the day and night, and sometimes more often (CYPS ward managers at South London and Maudsley NHS Foundation Trust, personal communication, 2023). This relates in part to potential for self-harm or adverse events relating to the mental health problem that led to admission. There is only one (very recent) study that has explored the barriers and facilitators to sleep for adolescent in-patients with psychiatric disorders, in a UK mental health unit. Interviews with 100 in-patients highlighted that more severe insomnia symptoms were associated with more severe affective and psychotic symptoms. Ward staff observed insomnia and excessive daytime sleepiness in the adolescent in-patients and a reciprocal relationship with mental health symptoms. Ward processes were barriers (e.g. night-time observations) and facilitators (e.g. regular routines) of sleep.^12^ We would argue that there is therefore a need to avoid sleep disturbance and promote regular sleep as part of treatment and recovery for in-patients.

### The safety of protected sleep periods

Frequently cited concerns about a more personalised sleep care plan relate to potential for harm overnight during a protected sleep period without nursing observations. Therefore, the monthly review of the standardised measures of all ward adverse events was important to highlight that personalised care plans were safe. There were no adverse events relating to the change in nursing policy. The regular opportunity to discuss and reassure staff in this regard was considered an important factor in the maintenance of the TeenSleepWell programme. There is no current evidence that a blanket policy of observations at fixed times is better than personalised care. Others have highlighted that a blanket overnight observations policy rather than a patient-focused sleep plan may be counterproductive and increase agitation.^5,13^

### Use of hypnotics

The review of hypnotics used on the wards was seen as an important outcome measure and one that reflected sleep and circadian rhythm disturbance requiring treatment. Previous work on the adult wards highlighted that knowledge of benefits and side-effects of hypnotics alongside education on behavioural strategies for insomnia reduced inappropriate prescriptions.^8^ On the CYPS units, the initial review of hypnotic use prior to starting TeenSleepWell highlighted that melatonin was the most widely used sleep aid and mostly used in the intellectual disability units. Zopiclone and promethazine were also used on the units. There remains little evidence for long-term benefit of promethazine for insomnia and, in addition, it has a relatively high side-effect profile.^6^ Although considered safer than traditional benzodiazepines, there remains a risk of tolerance and dependence with drugs such as zopiclone.^14^ Melatonin is used in children and adolescents with both circadian rhythm disorders and insomnia, but in the UK it is currently licensed only for those with autism spectrum disorder for whom behavioural interventions have been unsuccessful. Variable doses and timing are used, with few validated outcomes. This work highlighted the need for ward staff to review appropriateness and ongoing benefit when first issued. A more widespread regional review of melatonin prescribing is ongoing. The decrease in the use of zopiclone was encouraging, suggesting that better sleep on the units might have decreased the requirement for a hypnotic.

### Strengths and limitations

Strengths of this TeenSleepWell service improvement include the successful implementation of a multimodal sleep improvement package across a wide variety of CYPS units with different patient needs and including general adolescent, intellectual disability and secure units. Ongoing review of the trust's adverse events dashboards helped staff to understand the safety and benefits of a personalised overnight sleep care plan. This allowed all ward staff to improve their knowledge of sleep and sleep disorders, improve the ward environment and allow individual patients to have a protected 8 h sleep period, when assessed as safe. This has since been successfully incorporated into standard care and, to the best of our knowledge, this is unique within UK CYP mental health units.

Limitations include a lack of data showing when each patient started on TeenSleepWell as this was a service improvement project and not resourced to collect extra data, simply relying on ward sleep leads reporting at 3-monthly meetings. A transition to electronic prescribing also meant that it was not possible to link patients prescribed hypnotics with those having protected sleep and further work is underway to measure this specifically. Noise levels were not formally measured with decibel meters as this was outside the scope of the work. No formal qualitative analysis of feedback or economic analysis was performed. However, a cost saving was likely, given the known negative impact of poor sleep on mental health and the reduced use of hypnotic medications. Further work is required to see whether this programme affects duration of stay, ideally within a formal trial.

### Implications

We believe that protecting and improving sleep should be seen as a key part of in-patient care in those under 18 and so education on sleep and sleep disorders and assessment of noise and light levels on the ward is important for all staff. Additionally, a personalised sleep care and observation plan for CYPS patients should be a nursing standard alongside a dynamic risk assessment.

## Data Availability

Data are available on reasonable request.

## About the authors

**Kirstie N. Anderson** is a consultant neurologist and sleep specialist at Newcastle upon Tyne Hospitals NHS Foundation Trust and an honorary senior lecturer in the Institute of Neuroscience, Newcastle University, Newcastle upon Tyne, UK. At the time this work was carried out **Rod Bowles** was the Senior Nursing Lead for Positive and Safe, which leads risk and restrain reduction within Cumbria, Northumberland, Tyne and Wear NHS Foundation Trust, Newcastle upon Tyne, UK. **Christine Fyfe** is a pharmacist working in the CYPS units within Cumbria, Northumberland, Tyne and Wear NHS Foundation Trust, Newcastle upon Tyne, UK. **Ron Weddle** is Senior Nursing Manager and Deputy Director of Positive and Safe, Cumbria, Northumberland, Tyne and Wear NHS Foundation Trust, Newcastle upon Tyne, UK. **Patrick Keown** is a consultant psychiatrist and Associate Medical Director within Cumbria, Northumberland, Tyne and Wear NHS Foundation Trust, Newcastle upon Tyne, UK, and an honorary senior clinical lecturer in the Institute of Neuroscience, Newcastle University, Newcastle upon Tyne, UK.
